# Portable smartphone-integrated ratiometric fluorescence probe for visual detection of mercury ions in environmental water with greenness evaluation

**DOI:** 10.1039/d5ra06396e

**Published:** 2025-10-15

**Authors:** Kawan F. Kayani

**Affiliations:** a Department of Chemistry, College of Science, University of Sulaimani Qlyasan Street, Kurdistan Regional Government Sulaymaniyah 46001 Iraq kawan.nasralddin@univsul.edu.iq

## Abstract

Mercury is an extremely hazardous environmental pollutant with strong bioaccumulation potential, underscoring the need for rapid and sensitive detection of mercury ions (Hg^2+^) to protect both the environment and public health. In this work, a ratiometric fluorescence sensor was developed for the selective detection of Hg^2+^. The use of ratiometric fluorescence probes minimizes background interference, thereby enhancing accuracy compared to single-emission intensity-based nanoprobes. The sensor was fabricated by combining red carbon dots (r-CDs) with green carbon dots (g-CDs). The developed ratiometric probe exhibited high sensitivity, achieving a remarkably limit of detection (LOD) of 60 nM. Furthermore, the sensor's capability for visual detection was validated through a smartphone-based solution assay, with a LOD of 26.68 μM. To assess its environmental sustainability, three greenness assessment metrics AGREE, BAGI, and RAPI were employed, all confirming the method's outstanding eco-friendliness. Overall, the synthesized probe demonstrates strong potential for practical applications in the detection of Hg^2+^ in real samples.

## Abstract 2

This study developed a ratiometric fluorescence probe based on red and green carbon dots for the selective detection of Hg^2+^ and evaluated its environmental sustainability using AGREE, BAGI, and RAPI metrics.

## Graphical abstract

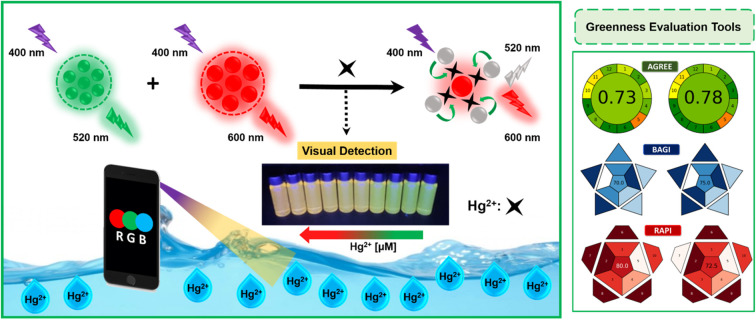

## 1 Introduction

Heavy metals have played a crucial role in the advancement of human civilization through their widespread industrial applications. However, their excessive use and the discharge of metal-contaminated wastewater have raised serious concerns regarding both human health and environmental safety.^1,2^ In particular, toxic heavy metal ions have attracted significant attention due to their harmful impact on ecosystems and the well-being of living organisms.^3^ The detection and monitoring of heavy metals in living cells and the environment are therefore essential to mitigate their adverse effects.

Mercury ions (Hg^2+^) are recognized as a highly toxic contaminant commonly found in soil, water, and food.^4,5^ Owing to their tendency to bioaccumulate, they can build up in living organisms and bind to thiol groups in proteins, leading to serious harm to the central nervous system and presenting a major risk to human health and the environment.^6–11^ Therefore, creating highly sensitive sensing approaches for the identification and detection of Hg^2+^ in environmental systems is essential. A range of methods, including ICP-MS,^12^ electrochemistry,^13^ SERS^14,15^ and AAS,^16,17^ have been proposed for Hg^2+^ detection. However, these traditional methods often suffer from drawbacks such as high cost, complex instrumentation, time-consuming sample preparation, and limited applicability in remote areas.^18,19^ In recent years, fluorescent sensors have emerged as promising candidates for heavy metal ion detection due to their high sensitivity, rapid response, affordability, and ease of signal detection.^20,21^ Therefore, fluorescence remains particularly significant for the rapid determination of Hg^2+^ in real samples.

To date, many fluorescence-based probes for the detection of Hg^2+^ have been developed, including polymers,^22,23^ DNA enzymes,^24^ metal nanoclusters,^25^ sulfur dots,^26,27^ covalent organic frameworks,^28^ metal organic frameworks,^29–31^ and semiconductor quantum dots.^32^ Most fluorescence-based sensors, however, depend solely on a single emission intensity as the responsive signal, which can be significantly affected by factors such as instrumental deviations, solvent effects, and more. In contrast to single-emission fluorescence methods, ratiometric fluorescence techniques can minimize these interferences and achieve higher analytical accuracy through the self-calibration of two fluorescence intensities. Typically, ratiometric fluorescence methods are accompanied by noticeable color changes, enabling rapid visual identification.^33–36^

Carbon dots (CDs) offer numerous benefits, such as low toxicity, cost-effectiveness, strong FL emission, excellent biocompatibility, chemical stability, and ease of synthesis.^37–40^ They have been widely employed as sensing probes for detecting a variety of targets, including metal ions,^41,42^ anions,^43^ and biological molecules.^44,45^ With the advancement of information technology, smartphones have become widely available. Recent research has explored their application as portable detectors for both colorimetric and fluorescence-based analyses. This strategy not only minimizes analysis time and expenses but also supports on-site and real-time testing.^46,47^

**Scheme 1:**
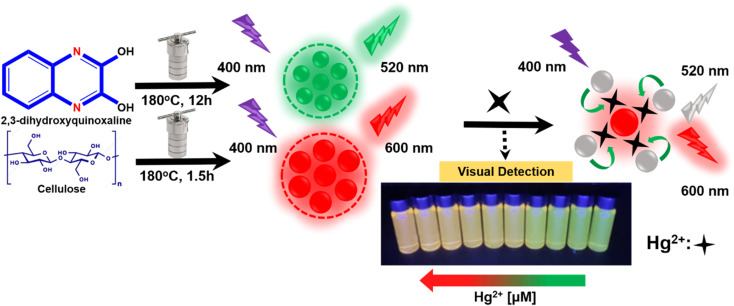
Illustration of the synthesis of CDs and the visual detection of mercury ions.

Here, as depicted in Scheme 1, a novel probe has been developed using green and red carbon dots (CDs) for the precise detection of Hg^2+^. With increasing Hg^2+^ concentrations, the green fluorescence of g-CDs at 520 nm was gradually quenched, while the red fluorescence of CDs at 600 nm remained unchanged. Additionally, the presence of Hg^2+^ could be visually detected through a distinct color change from green to red under UV light. This probe enables rapid, accurate, and efficient detection of mercury ions without the need for expensive or complex equipment, making it well-suited for environmental monitoring applications.

## 2 Experimental part

### 2.1 Synthesis of green CDs

The g-CDs were synthesized. Briefly, 0.2 g of 2,3-dihydroxyquinoxaline was dissolved in a mixture of ammonia, DMF, and deionized water in specific proportions (1.5 : 1.5 : 20 mL, respectively). The resulting solution was then transferred to a 50 mL autoclave and heated at 200 °C for 12 hours. After the heating process, the autoclave was allowed to cool naturally. The obtained solution was passed through a 0.22 μm microporous membrane to eliminate larger particles, resulting in a transparent filtrate. This filtrate was further purified *via* dialysis. After purification, the solution was stored at 4 °C for subsequent testing.

### 2.2 Synthesis of red CDs

The r-CDs were prepared following this procedure.^48^ 0.5 g of cellulose was dissolved in 40 mL of 2.5 M H_3_PO_4_ and heated at 180 °C for 1.5 hours. After that, the solution was purified.

### 2.3 Instruments

The optical properties of both CDs were characterized using a Cary Eclipse Fluorescence Spectrophotometer and a Cary 60 UV-Vis Spectrophotometer. Functional groups were analyzed by FTIR with a diamond ATR attachment (Nicolet iS50, Thermo Scientific). Morphology and structure were examined using high-resolution TEM (FEI Tecnai G2 F30), and surface composition was investigated *via* X-ray photoelectron spectroscopy (XPS, Thermo Fisher ESCALAB 250Xi).

### 2.4 The detection of Hg^2+^ with the probe

The ratiometric fluorescent probe was synthesized by combining g-CDs and r-CDs in a 2 : 1 FL intensity ratio in 2 mL of solution at pH 4. Hg^2+^ ions at varying concentrations were then introduced into the ratiometric fluorescent probe solution, and the resulting fluorescence spectra were recorded.

### 2.5 Visual detection

The solution containing a fluorescent probe (g-CDs and r-CDs in a 2 : 1 ratio) underwent the addition of different concentrations of Hg^2+^. Using a smartphone-based colorimetric device with a 365 nm UV lamp, we captured images of the fluorescence colors. The RGB values corresponding to the FL color information were then acquired utilizing the Color Grab Android application.^49^

### 2.6 Selectivity

To evaluate the specificity of the probe for Hg^2+^, 1 mM of the cations Co^2+^, Zn^2+^, Cr^3+^, Ni^2+^, Mg^2+^, Mn^2+^, Fe^2+^, Cd^2+^, and Cu^2+^, along with 1 mM of molecules such as glucose, ascorbic acid, phenylalanine, arginine, glycine, histidine, tartaric acid, and cysteine, were added to the system as potential interferences.

### 2.7 Real application

The fluorescence method was applied to detect Hg^2+^ in real samples, including laboratory tap water and commercially available bottled water from a local supermarket.

## 3 Result and characterization

### 3.1 Characterization of CDs

**Fig. 1:**
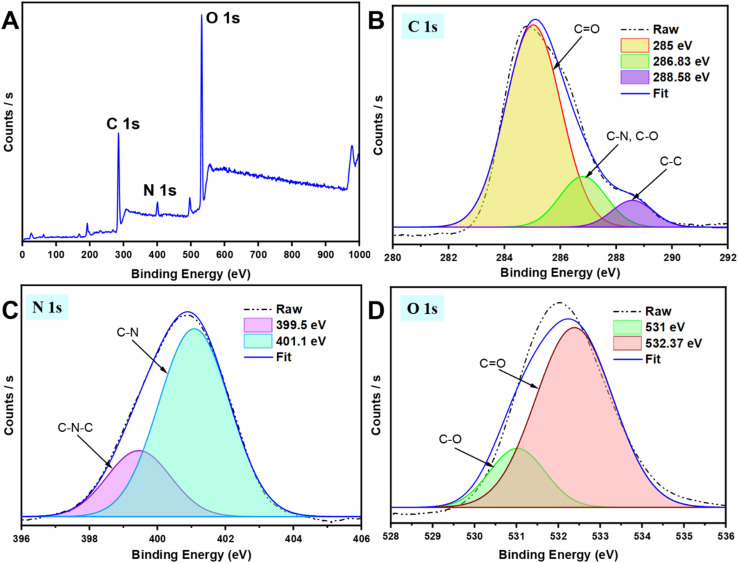
(A) XPS survey scan of g-CDs; (B)–(D) high-resolution XPS scans of the elemental composition of g-CDs.

The surface functional groups of g-CDs were further analyzed using XPS. As shown in Fig. 1A, the full-range XPS spectrum reveals three distinct peaks at 284.79, 398.81, and 532.49 eV, corresponding to the characteristic signals of C1s, N1s, and O1s, respectively. This suggests that the surface of g-CDs is primarily composed of nitrogen (N), carbon (C), and oxygen (O). Fig. 1B presents the high-resolution XPS spectrum of C1s, which shows three peaks associated with C=O, C–C, C–OH, and C–N functional groups. Similarly, the high-resolution XPS spectrum of N1s, shown in Fig. 1C, displays three absorption peaks corresponding to the characteristic signals of C–N–C and C–N groups.^50^ Additionally, as illustrated in Fig. 1D, the O1s spectrum features two peaks, which can be assigned to C–OH/C–O–C groups and C=O.^51^

**Fig. 2:**
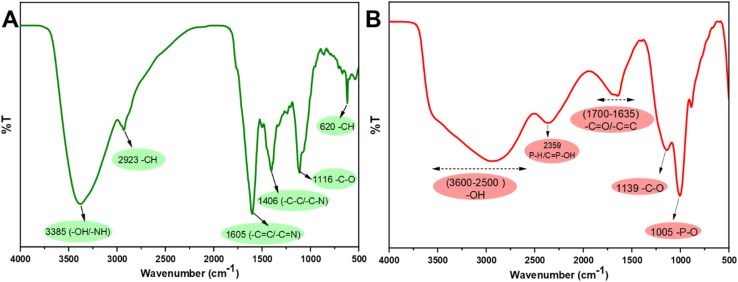
FTIR spectra of (A) g-CDs, and (B) r-CDs.

The FTIR spectrum of the g-CDs is presented in Fig. 2A. A broad absorption band at 3385 and 2923 cm^−1^ is attributed to O–H stretching and methylene or methyl (C–H) groups, confirming the presence of aliphatic hydrocarbons. The peak at approximately 1605 cm^−1^ corresponds to C=C stretching vibrations, while the bands at 1406 and 1116 cm^−1^ are assigned to C–N and C–O functional groups, respectively^52–56^ In addition, the spectrum of r-CDs is shown in Fig. 2B. The broad and intense band in the range of 3600–2500 cm^−1^ is attributed to O–H groups. The absorption peak at 2359 cm^−1^ corresponds to P–H groups. The broad peak at 1900–1635 cm^−1^ corresponds to C=O and C=C groups. Moreover, the band at 1139 cm^−1^ is related to C–O stretching vibrations.^48,57–59^

**Fig. 3:**
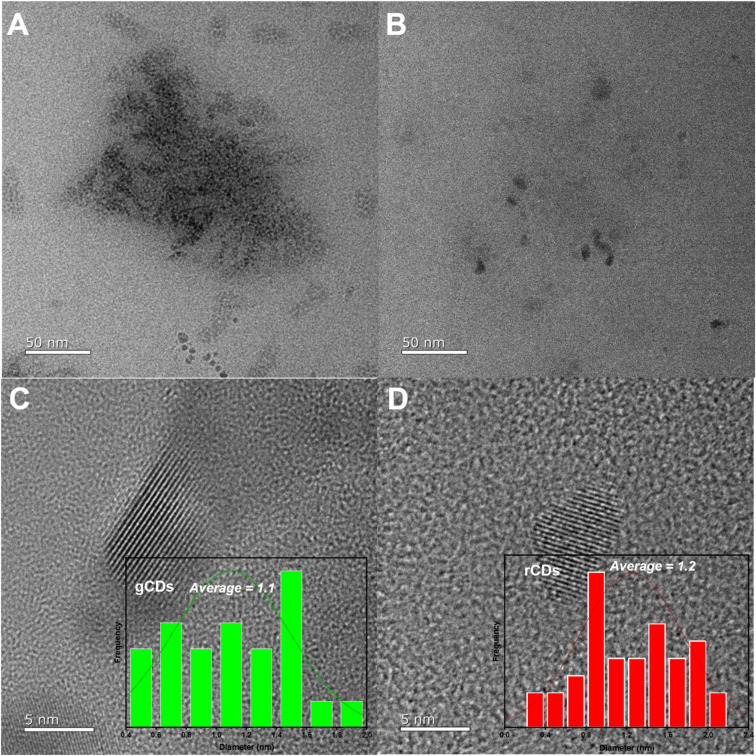
HRTEM images, and histogram distribution plot (A and C) g-CDs and (B and D) r-CDs.

The shape, size, and morphology of the green and red CDs (g-/r-CDs) were assessed by HRTEM. Fig. 3A and B show the TEM images and particle size distributions of both green and red CDs, which exhibit an approximately spherical shape. As illustrated in the HRTEM images, both g-CDs and r-CDs demonstrated excellent dispersion and uniform particle size. The average particle sizes were approximately 1.1 nm for g-CDs and 1.2 nm for r-CDs.

**Fig. 4:**
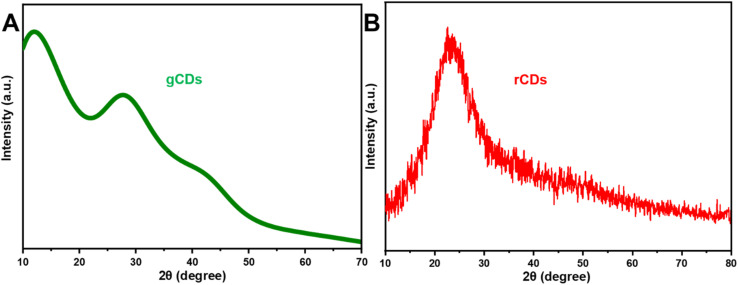
The XRD spectra of the prepared (A) g-CDs and (B) r-CDs.

The XRD patterns of g-CDs and r-CDs exhibit broad peaks, with the g-CDs pattern indicating the presence of highly disordered carbon atoms in the synthesized material (Fig. 4A). The g-CDs show broad 2*θ* peaks at approximately 27.6° and 42°, corresponding to disordered carbon atoms and confirming the predominance of the C(002) and C(100) planes associated with the hexagonal graphite structure of the g-CD particles.^60^ The broad XRD pattern suggests that the crystalline particles are small in size. Similarly, the XRD pattern of r-CDs (Fig. 4B) features a broad peak around 22.75°, indicating the presence of graphitic carbon (001) crystal planes within the r-CDs.^48^

### 3.2 Optical properties

**Fig. 5:**
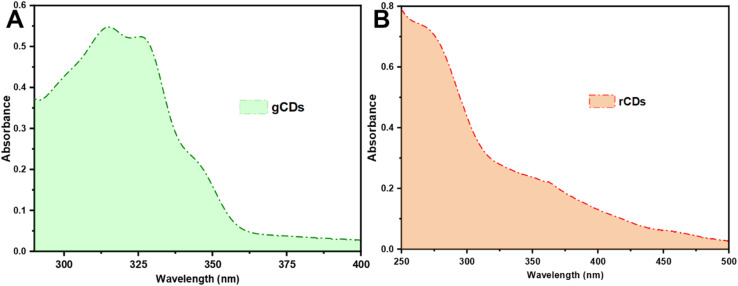
UV-Visible spectra of (A) g-CDs and (B) r-CDs.

The UV-Vis spectra of green and red CDs were examined. The UV-Vis spectrum of g-CDs, shown in Fig. 5A, displays a broad absorption band ranging from 275 to 360 nm. Notably, absorption peaks are observed at 276, 315, and 325 nm, corresponding to the π–π transition and the n–π transition, which are associated with aromatic sp^2^ hybridization and C=O double bonds, respectively.^60^ In contrast, the UV-Vis spectrum of r-CDs reveals an absorption peak at 275 nm and two additional peaks within the 330–500 nm range. The first peak is attributed to π–π* transitions, while the absorption between 330 and 500 nm is linked to n–π* transitions, as shown in Fig. 5B.^48^ Peaks observed beyond 300 nm are attributed to the presence of carbonyl-based groups on the surface.

**Fig. 6:**
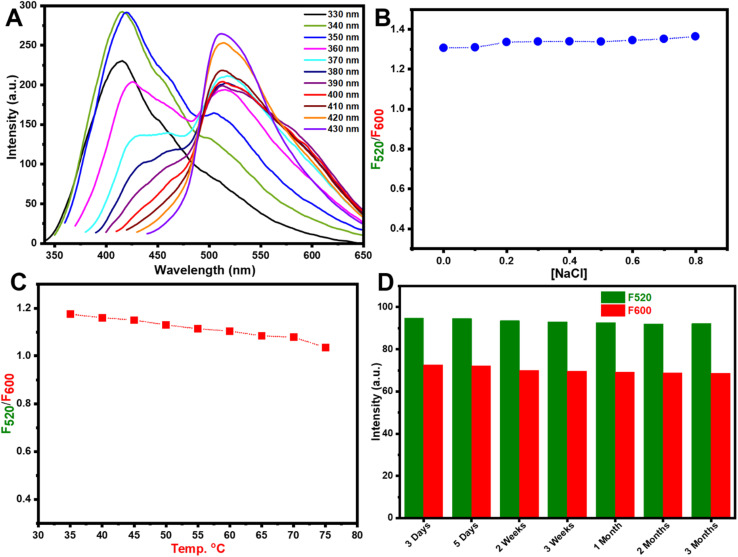
(A) Emission spectra of the ratiometric probe; (B) examination of IS; (C) effect of temperature; and (D) stability of the probe.

The dual-emission g/r-CDs fluorescence probe was easily created by combining green and reed CDs. Typically, dual-emission fluorescence probes exhibit two distinct emission peaks when excited by a single wavelength.^61^ As shown in Fig. 6A, the fluorescence emission intensity of g-CDs and r-CDs was optimal around the 390–400 nm excitation range. Based on Fig. 6A, 400 nm was chosen as the optimal excitation wavelength for the dual-emission g/r-CDs throughout the experiments.

### 3.3 Effect of ionic strength

The effect of ionic strength on the fluorescence intensity of the ratiometric system was investigated. As shown in Fig. 6B, the addition of NaCl had no impact on the fluorescence intensity of the system.

### 3.4 Effect of temperature

As depicted in Fig. 6C, the ratio of *I*_520_ to *I*_600_ remained relatively stable within the temperature range of 35–75 °C. Consequently, 35 °C was chosen as the optimal temperature for this study to facilitate ease of operation.

### 3.5 Stability test

The stability of the g/r-CDs was evaluated by monitoring their fluorescence intensity over a three-month storage period. As shown in Fig. 6D, no significant changes in fluorescence intensity were observed, indicating that the CDs maintained their structural integrity and optical properties with minimal variation, even after extended storage. This demonstrates the excellent long-term stability of the synthesized g/r-CDs, making them suitable for practical applications in environmental sensing.

### 3.6 Fluorescence detection

**Fig. 7:**
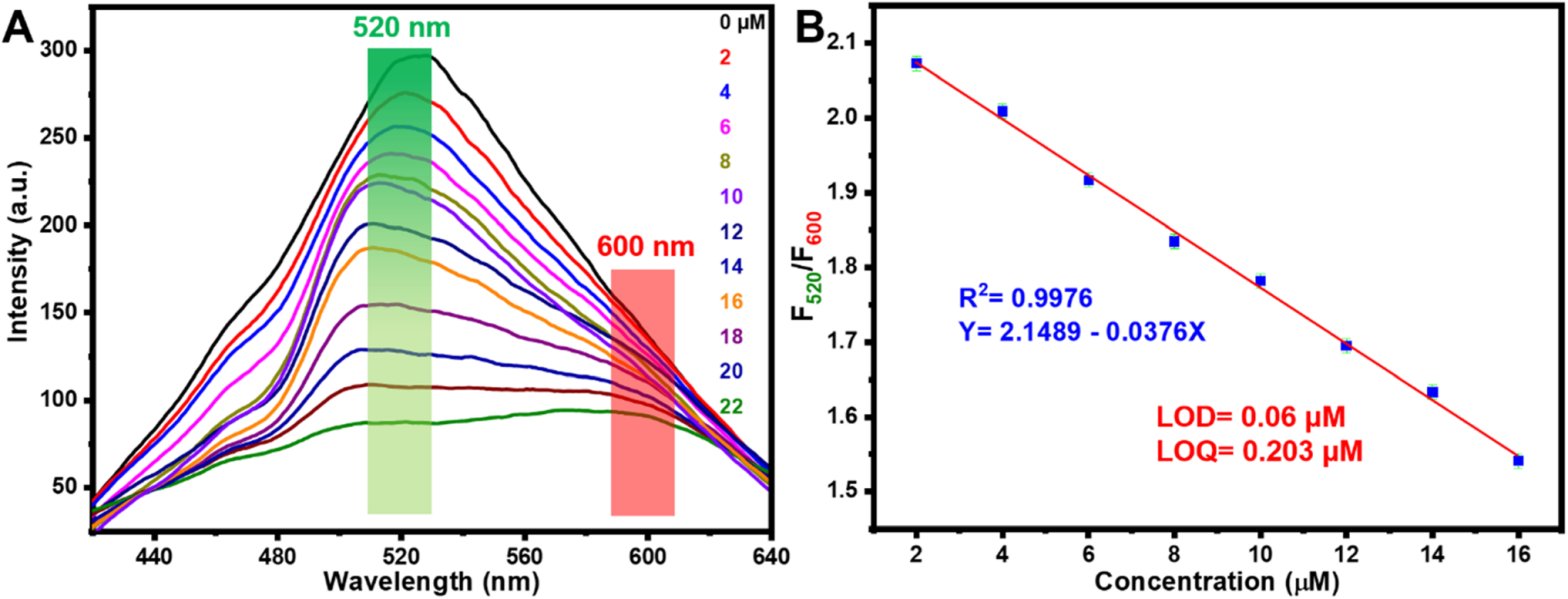
(A) FL spectra of green and red CDs with mercury ions (g-CDs and r-CDs in a 2 : 1 ratio). (B) Plot of *F*_520_/*F*_600_*versus* mercury ion concentrations (μM).

Fluorescence responses were recorded after the addition of varying concentrations of Hg^2+^ to assess the sensitivity of the ratiometric probe (Fig. 7A and B). As the concentration of Hg^2+^ increased, the fluorescence intensity of the green-emitting QDs gradually decreased, while the red-emitting CDs showed only a slight decline and served as reference signals. This variation in emission intensity between the two peaks resulted in a noticeable fluorescence color transition from green to orange and eventually red, enabling visual detection of Hg^2+^ with the naked eye. As shown in Fig. 7A, the fluorescence intensity ratio (*F*_520_/*F*_600_) exhibited a strong correlation with Hg^2+^ concentration. A linear relationship was established in the range of 2–16 μM, described by the equation *F*_520_/*F*_600_ = 2.1489 − 0.0376 × [Hg^2+^], with a high coefficient of determination (*R*^2^ = 0.9976). The LOD was determined to be as low as 0.06 μM (Fig. 7B).

As listed in Table 1, when compared with previous works on Hg^2+^ detection, this probe showed comparable performance in terms of fluorescence.

**Table 1:** Comparison of fluorescent probes used to detect Hg^2+^

Materials used	Sample	Linear range	LOD	Sensor type	Ref.
N-GQDs	Water	0–4.31 μM	23 nM	Turn-off	62
R6GH	Seafoods	0–5 μM	2.5 × 10^−2^ μM	Turn-on	63
BA-Eu-MOF	Water	1–60 μM	220 nM	Turn-on	64
SCDs	Water	0.05 to 5.8 μmol L^−1^	33.3 nM	Turn-off	65
NCQDs	Water	0–5.0 μM	0.017 μM	Turn-off	66
g/r-CDs	Water	0–16 μM	60 nM	Turn-off	This work

### 3.7 Selectivity investigation

**Fig. 8:**
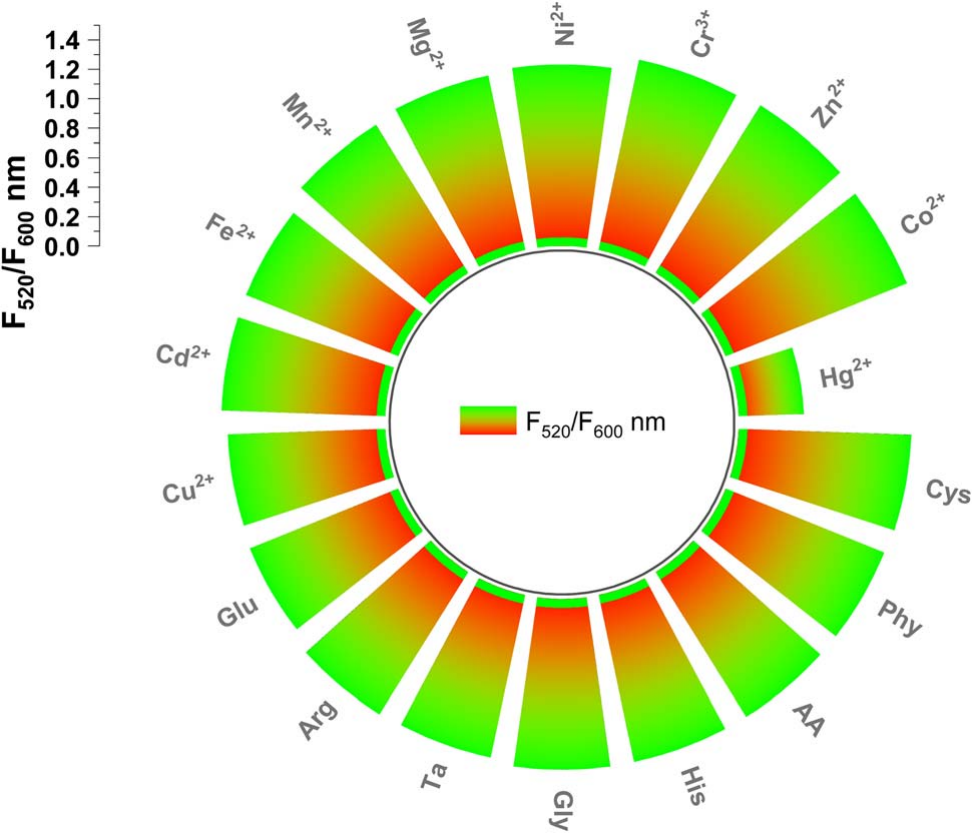
The selectivity study of the probe.

Selectivity is a key factor in assessing the detection capability of the ratiometric probe. To evaluate its specificity, the sensor's response was tested in the presence of potentially interfering ions. These interfering substances, including various metal ions and molecules, were introduced at concentrations of 100 μM ten times higher than that of Hg^2+^. As illustrated in Fig. 8, their presence caused only minimal changes in fluorescence, indicating no significant interference with Hg^2+^ detection. These findings confirm that the dual-emission ratiometric probe exhibits excellent selectivity for Hg^2+^. Furthermore, the sensor's performance remained unaffected even when Hg^2+^ coexisted with other ions.

### 3.8 Sensing mechanism

**Fig. 9:**
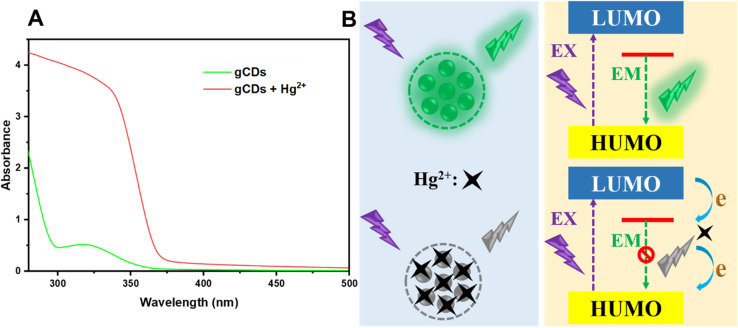
(A) The UV-Vis absorption spectra of g-CDs and g-CDs + Hg^2+^. (B) A schematic diagram illustrating the formation of blocked emissions in g-CDs.

The FL intensity of g-CDs at 520 nm was significantly quenched in the presence of Hg^2+^. This observation indicates that the synthesized g-CDs can function as a novel “on–off” fluorescent sensor for the sensitive detection of Hg^2+^. To investigate the sensing mechanism, the UV-Vis spectra of the sensing platform were analyzed. As depicted in Fig. 9A, no noticeable changes were observed when comparing the UV-Vis spectra of g-CDs alone and g-CDs with Hg^2+^. Additionally, no new peaks appeared in the sensing system, ruling out the possibility of static quenching (SQ). SQ is a common fluorescence quenching mechanism that alters the absorption spectrum through the formation of a ground-state complex. These results suggest that the quenching effect is likely caused by efficient electron transfer between g-CDs and Hg^2+^,^67^ To further elucidate the quenching mechanism of Hg^2+^ on the fluorescence of g-CDs, it is proposed that Hg^2+^ suppresses fluorescence through electron transfer between the surface ligands of the g-CDs and the Hg^2+^ ions.^55^ The remarkable selectivity of g-CDs toward mercury can be attributed to the preferential chelation of Hg^2+^ with oxygen and/or nitrogen atoms on the g-CD surface, a property not commonly observed with other metal ions. Experimental evidence from FTIR and XPS analyses indicates that hydroxyl and amine functional groups on the g-CDs readily coordinate with Hg^2+^, thereby providing high specificity. This strong interaction leads to the formation of a stable Hg-CD complex, which in turn results in fluorescence quenching,^68^ as illustrated in Fig. 9B.

### 3.9 Real application

The analytical performance of the ratiometric fluorescence probe for Hg^2+^ detection was evaluated using tap and bottled water samples, following the pretreatment procedures reported in the literature.^69^ As shown in Table 2, the recoveries of Hg^2+^ in real samples ranged from 96.84% to 105.72%, indicating that the probe is suitable for trace-level analysis of Hg^2+^ in environmental water.

**Table 2:** Determination of mercury in real samples (*n* = 3)

Sample	Spiked (μM)	Found (μM)	Recovery %	RSD (*n* = 3) %
Tap water	25	24.22	96.84	1.12
	30	30.09	100.27	2.03
Bottled water	25	26.44	105.72	2.45
	30	30.31	101	1.37

### 3.10 Visual based detection

**Fig. 10:**
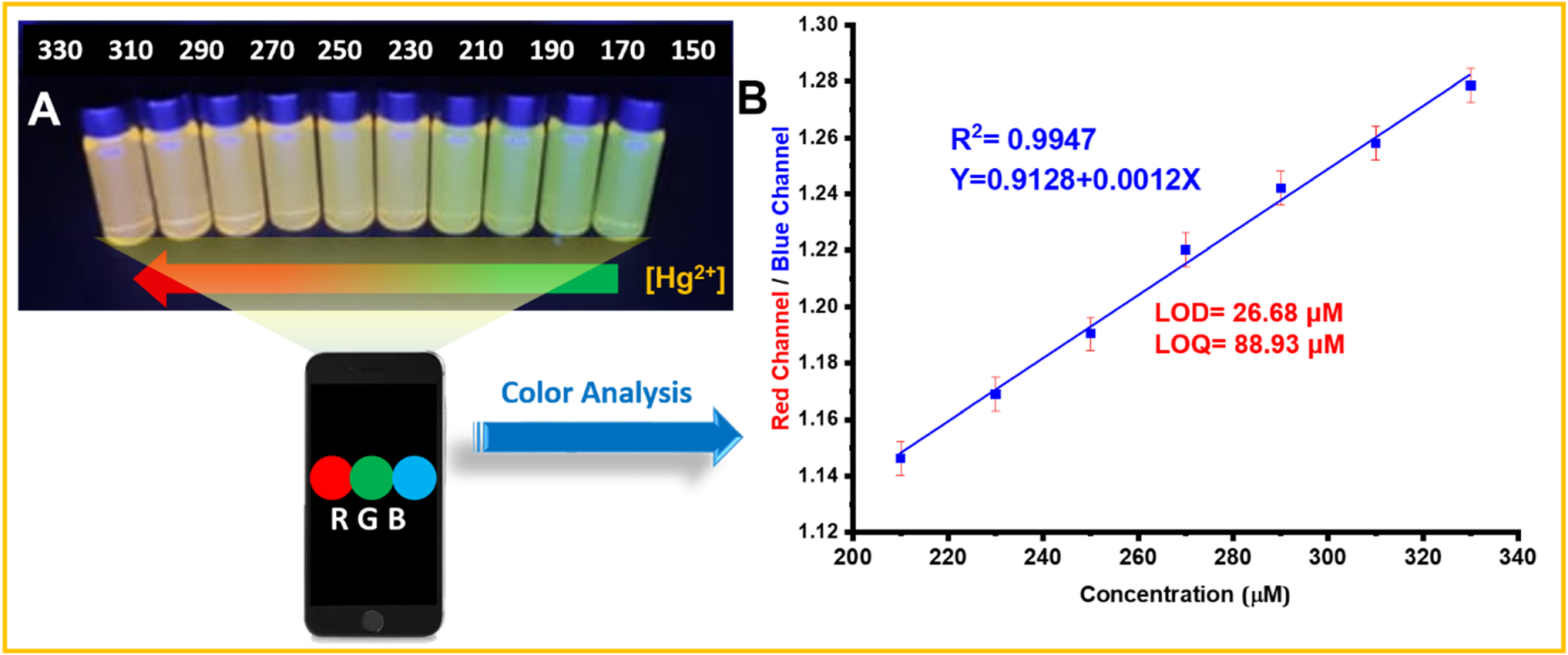
(A) Color changes observed in the solutions. (B) Calibration curve for the visual assay.

As the mercury concentration increased from 150 to 330 μM, a dramatic change in the visual color of the green and red CDs was observed, transitioning from green to orange and eventually to red under ultraviolet light (Fig. 10A). To extract the sample's color information (RGB values), the fluorescent image was separated into three channels using ImageJ. The results revealed that the fluorescence intensity in the red channel increased, while that in the blue channel decreased, with each channel displaying distinct linear ranges and sensitivities. By utilizing the ratio of red to blue channel intensities (*R*/*B*) as the signal, a linear regression curve was established to correlate Hg^2+^ concentration with the *R*/*B* values. In the concentration range of 150–330 μM, a strong correlation (*R*^2^) was observed between the *R*/*B* values and Hg^2+^ concentration, with a LOD of 26.68 μM and a LOQ of 88.93 μM (Fig. 10B).

### 3.11 Greenness profile

Environmental considerations are playing an increasingly important role in the development of analytical methods. To evaluate the eco-friendliness of both the sample preparation and the overall analytical process, three distinct assessment tools were applied.^60,70,71^ Specifically, two green evaluation tools AGREE and BAGI were used to assess the environmental impact of the sample preparation step as well as the complete analytical procedure.

**Fig. 11:**
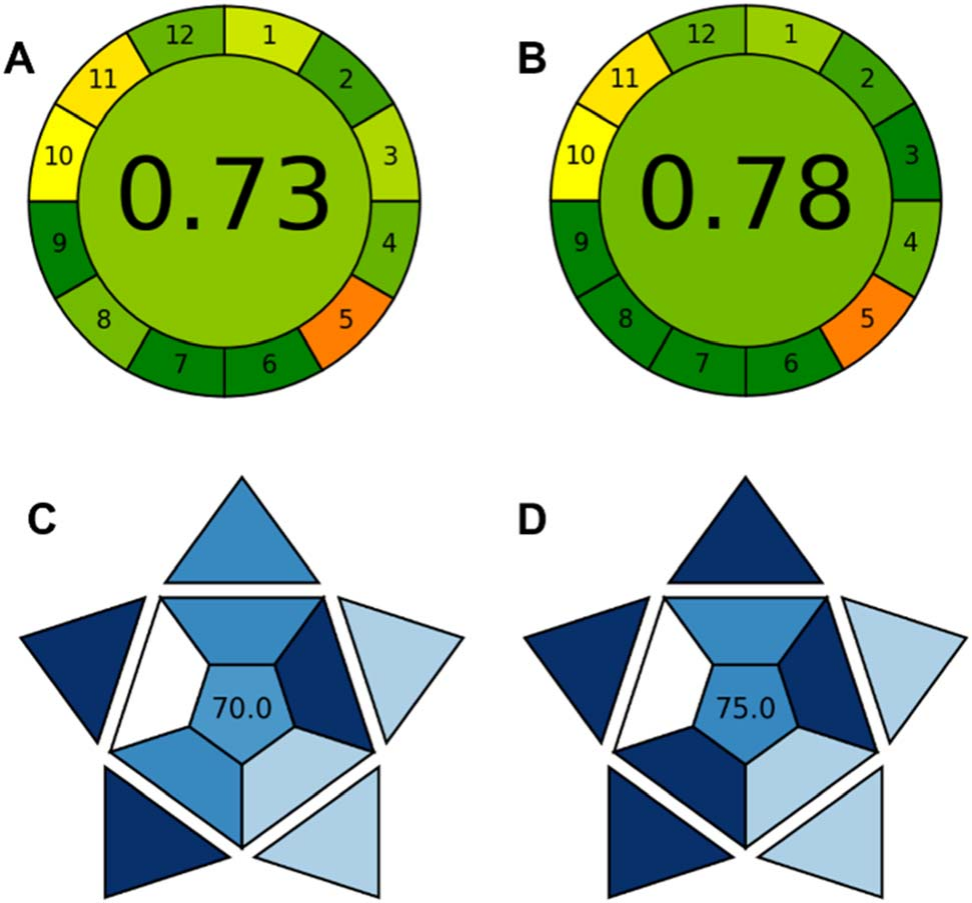
Evaluation of the environmental sustainability of the proposed fluorimetry (A) and smartphone-based (B) methods using the AGREE metric. Evaluation of the environmental sustainability of the proposed fluorimetry (C) and smartphone-based (D) methods using the BAGI metric.

The AGREE tool was used to assess the environmental sustainability of the two developed methods. As shown in Fig. 11A and B, both approaches demonstrated environmentally friendly attributes, with the visual based method achieving a higher score (0.78) compared to the fluorimetry method (0.73). This enhanced performance can be attributed to factors such as reduced sample volume, the ability to analyze multiple samples simultaneously, system miniaturization, and lower energy consumption. These findings highlight the safety, eco-friendliness, and environmental sustainability of the proposed techniques.

Furthermore, the BAGI tool was employed to assess the environmental impact of the methods involving fluorescent materials. As shown in Fig. 11C and D, the pictograms indicate BAGI scores of 75 and 70 for the visual-based and fluorimetry methods, respectively again underscoring the greener profile of the visual-based approach. Both methods also demonstrate strong potential for mercury ion detection.

**Fig. 12:**
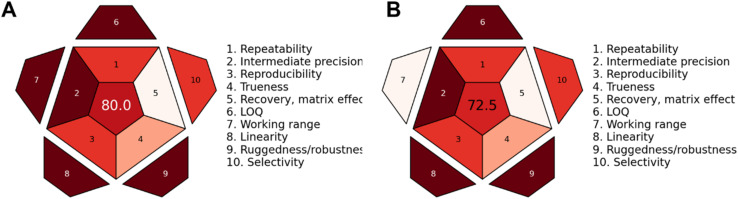
RAPI analysis: (A) fluorimetry, (B) smartphone-based approach.

Another novel tool introduced by Nowak *et al.*, the Red Analytical Performance Index (RAPI), was used to evaluate the proposed method.^72^ This tool is applied here for the first time in a CD-based sensing approach and considers 10 parameters to evaluate analytical performance. Based on these 10 criteria, the fluorimetry method achieved a score of 80 (Fig. 12A), while the smartphone-based approach scored 72.5 (Fig. 12B). The lower score of the smartphone-based approach is primarily due to its working range, which is not as strong as that of the fluorimetry method.

## 4 Conclusions

In conclusion, the dual-wavelength fluorescent probe features an inherent self-calibration capability, which significantly enhances both the sensitivity and accuracy of detection. This strategy capitalizes on the advantages of ratiometric fluorescence techniques, enabling semi-quantitative visual detection of Hg^2+^ and accurate quantification through dual-channel fluorescence signals. The sensor demonstrated excellent sensitivity toward Hg^2+^, achieving low detection limits. Moreover, it was successfully applied to the rapid analysis of Hg^2+^ in real samples, yielding high accuracy and satisfactory recovery rates. These findings suggest that the proposed fluorescent probe has strong potential for use in environmental monitoring and analytical sensing applications.

## Conflicts of interest

There are no conflicts to declare.

## Data Availability

The authors confirm that the data supporting the findings of this study are available within the article.
